# A qualitative research on emotion regulation processes and expressive language skills in kindergarten: a case study

**DOI:** 10.3389/fpsyg.2025.1611554

**Published:** 2025-06-23

**Authors:** Sena Nur Oğuz, Yunus Pınar

**Affiliations:** Department of Early Childhood Education, Akdeniz University, Antalya, Türkiye

**Keywords:** emotion regulation, emotional development, expressive language skills, observation according to Tavistock model, videography

## Abstract

This single-case study focused on Aylin (name anonymized), a typically developing five-year-old girl, observed over 9 months following her kindergarten enrollment. Using the Tavistock Child Observation Model, videography, HAVAS 5 language screening, and semi-structured parental interviews, the study examined her social interaction, emotion regulation, and expressive language development. Aylin experienced marked anxiety and stress, especially during the adjustment phase, struggling with separation from her parents and the unfamiliar school environment. Initially withdrawn, she gradually developed strategies to engage with peers and seek inclusion in group activities. Emotional security was a key factor in her ability to play cooperatively and express herself more fluently. HAVAS 5 results reflected notable progress in her expressive language skills by the end of the year. Parental interviews revealed that Aylin had spent over a year in pandemic-related isolation starting at age two and a half, limiting her social and physical development. Both parents cited challenges in offering consistent interaction, which may have shaped Aylin’s early difficulties. The findings underscore the interconnectedness of emotional regulation, symbolic play, and language expression, emphasizing the value of emotionally responsive early education.

## Introduction

Children who are able to regulate their emotions tend to deal with conflicts more constructively and foster supportive, positive relationships. Optimal emotion regulation in early childhood is associated with greater psychological resilience (Polizzi and Lynn, 2021), psychological well-being (Kraiss et al., 2020), and academic success (Blair, 2002; Liew et al., 2019; Kwon et al., 2017; Wong et al., 2023). It also plays a crucial role in shaping peer relationships. Children who struggle with emotion regulation are at greater risk of social rejection, isolation, and antisocial behavior in later years (Blair et al., 2004; Séguin and MacDonald, 2018; Vitiello et al., 2022). Numerous studies across disciplines have examined the development of emotion regulation using various methodologies. For example, Galyer and Evans (2001) found that children engaged in frequent pretend play with caregivers exhibited stronger emotion regulation. Similarly, Cohen and Mendez (2009) found that children with aggressive or disengaged peer play behaviors showed less social improvement across the school year. Research also shows that emotion regulation develops prior to and predicts emotion knowledge (Lucas-Molina et al., 2020) and is positively influenced by fantasy-oriented play (Gilpin et al., 2015). Other studies link emotion regulation to variables such as role-play (Hoffmann and Russ, 2012), emotional knowledge (Di Maggio et al., 2016), peer dynamics (Ogelman and Fetihi, 2021), attachment (Volling, 2001), social competence (Hamaidi et al., 2021), and executive functions (Fuster et al., 2009). While quantitative research has advanced the field significantly, ethnographic studies exploring emotion regulation as a dynamic, contextualized process are gaining importance. Observational approaches based on the Tavistock model (Bick et al., 2018) offer micro-analytic insights into emotional development. Key contributions include the work of Diem-Wille (1997), Briggs (1997), Davenhill (2018), Rustin (2011a, 2011b), Lazar (2000a), and Elfer (2012, 2014).

At the University of Vienna, Tavistock-based observation has informed several longitudinal studies exploring transitions to out-of-home care (Datler et al., 2010), premature infant development (Spatz, 2004), early intervention for children with disabilities (Datler and Isopp, 2004), and the link between emotion regulation and language development (Datler et al., 2014a).

In early childhood research, it has been widely proposed that as children’s language abilities mature, these skills begin to serve as tools for emotional regulation—allowing children to label, communicate, and eventually manage affective states through verbal means (Cole et al., 2010). However, despite this compelling theoretical linkage, the developmental pathways through which language facilitates emotion regulation remain underexplored in empirical literature (Roben et al., 2013). Existing longitudinal studies suggest that children with stronger and faster-developing language skills exhibit lower levels of anger expression over time and are more likely to engage in adaptive strategies such as support-seeking and distraction (Reilly and Downer, 2019; von Salisch et al., 2015). Yet, these studies often rely on structured assessments that may overlook the relational and symbolic textures through which language and affect co-develop in everyday contexts. This case study seeks to address this gap by observing how a child’s expressive language unfolds in tandem with emotion regulation processes across a full year of naturalistic interaction. Through fine-grained analysis of symbolic play, narrative behavior, and affective expression in context, the study examines not only whether but how language becomes a vehicle for emotional self-regulation (Pahigiannis and Glos, 2020). As such, it contributes to a growing body of work that views language, play, and emotion not as isolated domains, but as mutually constitutive elements of the developmental ecology of early childhood (Gilpin et al., 2015; Monopoli and Kingston, 2012; Ornaghi et al., 2020).

### The educational context in post-pandemic Turkey

This study was conducted in a preschool setting in Turkey following the COVID-19 pandemic. As a global event that profoundly disrupted the developmental ecologies of early childhood, the pandemic significantly altered the normative course of young children’s social and emotional experiences. In Turkey, prolonged school closures, restrictions on social interactions, and limited access to outdoor and peer-based play environments constrained children’s opportunities to develop foundational social competencies and emotion regulation skills. According to insights gained through face-to-face interviews with the focus child’s parents, the pandemic overlapped with a sensitive developmental period shortly after her second birthday—curtailing both family bonding experiences and exposure to diverse social contexts (see Supplementary Excerpt 40). While Turkish early childhood education is culturally rooted in emotionally attuned and relationally warm adult-child interactions, institutional practices often lean toward structured, adult-centered routines. This tension became even more pronounced in the aftermath of the pandemic, as children like Aylin returned to educational settings with heightened emotional needs and fragile social confidence. The present study is thus situated within a pedagogical landscape shaped by the intersection of systemic rigidity and the urgent need for relational sensitivity—an ecology that informs not only Aylin’s transition but also the emotion regulation and expressive language processes explored throughout this case.

### The current study

The latest research in neuroscience and developmental psychology demonstrates how emotions play a key role in influencing human behavior and cognitive processes (Damasio, 1999; LeDoux, 2003). Even though Lüdtke (2012) describes a growing focus on emotion within scientific disciplines as the “emotional turn,” research on emotion regulation among young children has yet to expand significantly. Particularly scarce are studies that use ethnographic and psychoanalytically informed observation methods to explore these processes in depth. This single-case study focuses on a typically developing five-year-old girl and aims to examine, over the course of a kindergarten year, how she experienced social interaction, emotion regulation, and expressive language development. The study employed naturalistic observation based on the Tavistock model, videography, and interviews to investigate how emotional and linguistic competencies evolved in relation to her environment. By integrating multiple methods, the study seeks to offer a detailed account of emotion regulation as a dynamic, relational, and embodied process in early childhood.

This study adopted a triangulated research design combining naturalistic observation, videography, and in-depth parental interviews, each selected for its capacity to illuminate different layers of the child’s developmental experience. The kindergarten environment was considered the child’s natural setting and served as the primary context for observing emotional and social behavior as it unfolded in real time. Videography was employed not merely as a supplementary method but as a tool for methodological cross-validation—enabling the identification of moments that confirmed, deepened, or productively challenged the insights derived from field notes. Its inclusion enhanced the reliability and granularity of the observational data. Meanwhile, parental interviews were conducted to reconstruct the emotional ecology of the home: to understand how the child experienced daily life outside the classroom, what forms of interaction she shared with her caregivers, and what emotional textures shaped her early symbolic world. By integrating these three perspectives, the study sought to construct a developmentally grounded, ecologically valid account of how emotional regulation and expressive language evolve in the interplay between home and school.

### Research questions

1 What kinds of social interaction experiences did the focus child have with peers and teachers during the kindergarten year?
2 What developmental changes occurred in the child’s emotional life, and how did she experience processes of emotion regulation?
3 What types of changes and progress were observed in the expressive language skills of the focus child during the observation process?
4 What insights can be gained about the focus child’s daily life outside of kindergarten, and how do her parents describe their thoughts and feelings about her?

While the study articulates four distinct research questions, they are conceptually interwoven and collectively serve a unified developmental inquiry. Each question captures a discrete dimension of the child’s early experience—namely, her social interactions, emotional regulation, expressive language skills, and family context. Taken together, they aim to construct a holistic understanding of how emotional and communicative capacities unfold and interact within the relational ecosystem of early childhood. This integrated perspective underlies the study’s methodology and analytical approach.

### Process

To address the concerns of school administrators and ensure transparency, meetings were held with several kindergarten principals. During these meetings, the research team explained that video recordings would be conducted in a discreet and non-intrusive manner, used exclusively for research and, when applicable, teaching purposes. It was emphasized that only the research team would have access to the data, that all personal identifiers would be removed or obscured using anonymization techniques such as blurring or mosaicking, and that under no circumstances would the data be used for commercial purposes. It was also made clear that participants had the right to withdraw their consent or request the destruction of audiovisual material at any point during the research process. Although most schools declined participation for various reasons, one kindergarten principal granted full approval. The study was carried out in that setting, with full adherence to ethical and institutional guidelines (Heath et al., 2010). Only one kindergarten granted approval for the videography component of the study. In this setting, written consent was obtained from the school principal, the classroom teachers, the parents of the focus child, and the legal guardians of all children who might appear in the recordings.

### Anonymization

The names of the participants mentioned in this study are anonymous and do not reflect their actual identity. The names were decided at the first seminar meeting of the project team and these names were chosen at random. The following pseudonyms are used to refer to people who are frequently mentioned in the study:

Focus Child: Aylin.

The father of the focus child: Mr. Murat.

The mother of the focus child: Mrs. Yeliz.

The teacher of the focus child: Mrs. Burcu.

The class teacher of the 6 year old: Mrs. Sema.

German teacher: Mrs. Hilal.

### Design and method

This study is a case study, which is a research method in which a specific event or situation is analyzed in detail. In this study, methods such as observation, videography and interview were used and HAVAS 5 (profile analysis approach applied to oral discourse) and demographic information form were employed as data collection tools. In this study, observation protocols, video transcripts and transcriptions of face-to-face interviews were analyzed using depth hermeneutics (Bereswill et al., 2010).

### Young child observation according to the Tavistock model

This study employed the Tavistock model of young child observation, an adaptation of Esther Bick’s original infant observation method (Bick et al., 2018; Diem-Wille, 2013). Widely used in psychoanalytic and educational research, this method enables detailed examination of children’s emotional development, affect regulation, social interactions, and inner fantasy life (Datler et al., 2014a; Datler et al., 2018).

One non-participant observer conducted weekly, hour-long observations of the focus child over an entire school year, documenting behavior and interaction in rich, chronological protocols. These were later discussed in weekly seminar sessions guided by a supervisor. This group setting served as a reflective space to explore both manifest behavior and underlying emotional dynamics, enhancing observational sensitivity and interpretive depth (Trunkenpolz et al., 2010).

The method’s strength lies in its capacity to access empirical data on subtle relational processes that are often inaccessible through questionnaires or structured interviews (Datler et al., 2014b). Through regular observation and detailed reflection, this approach provides insight into the evolving emotional life of the child and the meaning of her behaviors in context.

In this study, the observer conducted 26 individual observations of Aylin from September 2022 to June 2023, and each session was followed by seminar discussions. Reports from these seminars and interim analyses informed the later case interpretation. Countertransference reactions were also considered as part of the reflective process, in line with depth hermeneutics (Bereswill et al., 2010).

### The analysis of the observation protocols

The interpretation of the observation protocols was guided by four key assumptions drawn from psychoanalytically informed observation literature:

1 Behavior as Expression of Inner Experience: The observed behaviors—both verbal and non-verbal—were viewed as reflections of the child’s conscious and unconscious emotional life. Understanding Aylin’s daily behavior in kindergarten required attention to her inner emotional states.
2 Observer Resonance: The observer’s own emotional and psychological reactions during the observation were considered meaningful. It was assumed that Aylin’s behavior could evoke emotional resonances—both conscious and unconscious—within the observer.
3 Interpretive Verification: To minimize misinterpretations shaped by the observer’s personal assumptions or projections, each observation was cross-checked by connecting the observer’s inner responses to the manifest behaviors described in the protocol. This ensured both a deeper understanding of the child and a self-reflexive stance from the observer.
4 Group Reflection: These observations were regularly presented in seminar settings, where they were interpreted collectively. The group discussions offered alternative perspectives, uncovered overlooked dynamics, and functioned as a mechanism for communicative validation, helping to refine and correct individual interpretations.

These methodological foundations align with established literature on infant and child observation using the Tavistock/Bick model (Lazar, 2000b; Datler et al., 2014b).

### Videography

Videography refers to an interpretive method for analyzing verbal and non-verbal communication in natural contexts, focusing on how interaction unfolds in real time (Tuma et al., 2013; Tuma and Schnettler, 2019). As defined by Knoblauch et al. (2006), it combines ethnographic fieldwork with video analysis to capture and interpret social interactions *in situ*. The researcher collects audiovisual data while being present in the field, making videography distinct from video interpretation (Knoblauch, 2011).

In this study, videography was used alongside Young Child Observation (YCO) based on the Tavistock model to enable methodological triangulation. Triangulation—using multiple methods or data sources—enhances validity by comparing findings across approaches (Denzin, 1970). Observation protocols and video data were cross-validated to reinforce interpretations.

Among the available analysis strategies in videography, this study employed sequence analysis to explore emotionally or socially significant moments in detail. This method was selected for its focus on the temporal and interactional unfolding of events (Dinkelaker and Herrle, 2009), allowing for in-depth microanalysis of selected scenes.

### Transcription of video recordings

In this study, the EXMARaLLDA Partitur Editor program was used to transcribe the video recordings and the researcher received three separate training sessions from the expert in order to become competent in the use of this program. Transcription of each video took approximately 18 h, resulting in a total of 234 h of transcription. The transcript of all the videos comprises approximately 550 pages and is kept as grey literature. Where necessary, it is considered appropriate to share them with requesting institutions in accordance with legal requirements. Audio-visual data and transcripts are stored on SD cards (Secure Digital Memory Card) and cloud storage files (locked virtual data repositories); a copy of the transcripts is also stored in folders.

### Interviews

The present study conducted semi-structured face-to-face interviews with the parents of the focus child, Aylin, to obtain information about her daily life outside of kindergarten. Two interviewers took part in this part of the study. The interviewers asked the mother and father questions in separate interview rooms at the same time, using questions that had been prepared in collaboration with experts. Both interviews lasted approximately 1 h and audio recordings were made during the interviews. At the end of the interview, the audio recordings were transcribed using EXMARaLDA Partitur and folded for inclusion in the grey literature. Finally, the data from the interviews were interpreted hermeneutically. Parents were informed that audio recordings would be made, and the audio recordings were not shared with anyone other than the researcher and supervisor. The right to share the audio files and transcripts with experts is reserved if this is deemed necessary or if it is required for scientific purposes.

### Demographic information form

This is a form that is given to the parents or caregivers of all children aged 60–72 months attending the kindergarten in order to identify the focus child. The form, which was developed in collaboration with experts for this purpose, includes questions about the child’s age, sex, whether the child has any developmental problems, and the age, occupation, educational status and average monthly income of the parents or caregivers.

### HAVAS-5

HAVAS-5 is administered in an interview format designed to assess expressive language skills in 5- to 7-year-old children. During the session, children are shown a six-frame picture story titled *The Cat and the Bird* and are encouraged to describe what they see in their own words. The visual narrative serves as a prompt to elicit spontaneous speech and narrative competence.

The story follows a simple but engaging plot involving a bird and a cat. In the initial sequence, the cat attempts to catch the bird, who is peacefully perched on a wall. As the story unfolds, the bird escapes to a tree, and the cat follows in pursuit. Eventually, the bird evades the cat’s reach and returns to safety, while the cat ends up distressed and alone.

Throughout the task, children are prompted with questions to encourage elaboration, and their responses are audio-recorded for later analysis. Narrative quality, vocabulary, grammar, sentence structure, and communicative engagement are all considered in the scoring. The test offers a standardized yet open-ended way to evaluate expressive language, allowing insight into the child’s verbal fluency, syntactic ability, and emotional engagement with symbolic content (Reich and Roth, 2007).

During the interview, children are encouraged to describe the story in detail, using guiding questions such as “What is the cat doing here?” or “What do you think the bird is feeling here?” Audio recordings of the interviews are transcribed and analyzed using a standardized evaluation form. The analysis focuses on five key dimensions: task performance, communicative competence, vocabulary, grammar, and sentence formation. These subcategories provide a framework for assessing both the structural and expressive components of children’s language. Originally developed for use in Hamburg primary schools in 2003, the Havas-5 diagnostic tool is available in multiple languages, including German, Turkish, Italian, Russian, Portuguese, and Polish (Reich and Roth, 2004; Stampf, 2012).

### Findings

In the following section, interpretive insights are drawn from longitudinal observation data. To support intersubjective comprehensibility, all interpretive claims are grounded in widely recognized developmental frameworks and substantiated with detailed observational excerpts. This approach ensures that interpretations are not merely subjective impressions, but analytically transparent and accessible to a broader scholarly audience.

### Findings from the analysis of observation protocols

This section presents an interpretative analysis of the protocols from the Tavistock child observation study, which convey certain aspects of the experiences of the focus child, Aylin, during a kindergarten year. Answers will be sought to the questions of what kind of social interaction experiences the focus child Aylin had with her peers and teachers from her first month in kindergarten, how she experienced daily life in kindergarten in the following process, and what kind of changes and differences emerged in her emotional world and emotion regulation processes.

### First month: the transition-adjustment process and the emotional atmosphere of the kindergarten

#### First month: transition and emotional atmosphere in kindergarten

The observer arrived at the kindergarten at 08:00 and documented children’s arrival experiences. During this period, the separation between children and their parents created a notably intense and distressing emotional atmosphere. Two separation episodes—one involving a 5-year-old and another a 2-year-old—revealed parallel expressions of distress: crying, protesting, clinging to the parent, and escalating emotional collapse, especially when caregivers used dismissive or frustrated language (see Supplementary Excerpts 1, 2).

Aylin’s own arrival was marked by visible distress. She silently cried while avoiding eye contact and attempted to hide her tears—suggesting emotional withdrawal and a coping strategy rooted in resignation. Although it remains unclear who dropped her off, the observer noted that Aylin exhibited signs of sadness and exhaustion following the separation (see Supplementary Excerpt 3). Her restrained crying and effort not to be seen reflected an attempt to manage an overwhelming moment internally (see Supplementary Excerpt 4).

Upon entering the classroom, Aylin was sent to the sink without verbal acknowledgment or emotional support from the teacher. This response ignored the emotional residue of the recent separation. In this moment, Aylin was required to manage two simultaneous tasks: processing the pain of leaving her caregivers and adapting quickly to a new, socially complex environment filled with unfamiliar adults and peers.

Another observation of a three-year-old in distress during separation reveals similarly intense emotional disorganization, with escalating sobs met by ineffective or even anxiety-inducing responses from the parent (e.g., threats not to return). Teachers were observed to lack effective strategies for co-regulating such distress in children (see Supplementary Excerpt 5).

### Attempts by the focus child to make contact with peers

Observation protocols from the first month revealed that Aylin continued to attempt to contact her peers using a variety of tactics. On the basis of the initial observation protocols, it is assumed that Aylin has a strong desire to have different interaction experiences with her peers and to play with them. Aylin tries various tactics to make contact with her peers. A detailed observation of Aylin’s imaginative play and her subtle attempts to engage with peers during the first month is included in the Supplementary Excerpt 6.

The observer describes what Aylin is holding in her hand as an “Imaginary liquid.” However, in discussing this section in the seminar meeting, the idea was floated that what Aylin was holding in her hand might actually be ‘Tea’. This idea helped the observer broaden her perspective and interpret Aylin’s subsequent behaviors in a more relational and symbolic context (see Supplementary Excerpt 7).

The interpretation that Aylin was preparing and serving tea is reinforced by her continued actions, which involve organizing toy cups and coasters in a careful and intentional manner, as illustrated in Supplementary Excerpt 8.

Even when Aylin is rejected by her peers, she shows the courage to develop a Plan B and try alternative strategies. She carefully places the cups and coasters on the table and begins making tea again. After a while, she attempts to balance the cups on her arm due to the absence of a tray—intending to bring them to the two girls playing nearby. The fact that Aylin prepares exactly three cups may suggest that she wants to join the other two girls and experience a sense of togetherness.

### Overview of the first month of observation

The first observation took place on 28 October 2022, followed by five additional sessions over the next month. During this period, it became evident that Aylin, the focus child, struggled with the separation process during drop-off. She was sometimes seen holding back tears and at other times quietly crying, suggesting that the transition into kindergarten was emotionally challenging for her.

In both the classroom and the garden, Aylin often adopted a passive stance. She avoided verbal communication, and her interaction attempts were limited to vague, nonspecific gestures. She mostly wandered aimlessly, observing her peers from a distance. These patterns indicate that she had difficulty adjusting to the new environment, likely due in part to the absence of a structured orientation process during her initial weeks.

When her efforts to engage with others were unsuccessful, Aylin tended to withdraw, but moments of brief joy—like a small smile—suggested emotional fluctuations. Her repeated attempts to join peer activities despite setbacks showed a persistent desire for social connection and belonging.

### The second and third months of the observation period

#### Aylin’s repeated attempts to connect with her peers

During the second and third months, Aylin continued to seek connection with her peers but often faced subtle rejections. In one instance, she carefully built three identical Lego figures and approached two girls, Nil and Esra, offering them as a gesture of inclusion. Echoing her earlier “tea-serving” attempt from the first month, this effort was again declined. Aylin’s quiet withdrawal and restrained body language indicated disappointment (see Supplementary Excerpts 9, 10). The observer noted that after this rejection, Aylin deconstructed the three figures and rebuilt them as two—possibly symbolizing the exclusion of herself. This act reflected an internalized sense of being unwanted, suggesting emotional vulnerability and self-effacement.

In another scene, the class was preparing a pretend party organized by Duygu. Aylin observed quietly, adjusting her proximity to the group without verbal participation. Her muted efforts to be included seemed unnoticed. When Duygu later offered her a toy, Aylin declined, possibly signaling emotional withdrawal after repeated setbacks (see Supplementary Excerpts 11, 12). Aylin’s final attempt—offering a red Lego as a self-made “ticket” to the party—was a symbolic gesture of longing and quiet resignation. When Duygu responded with, “You were supposed to get it from me, but never mind,” Aylin’s smile faded. Her blank gaze and stillness conveyed silent disappointment. The observer poignantly remarked: “I am almost sure her heart is in pieces” (see Supplementary Excerpt 13).

### Overview of the observations for the 2nd and 3rd month

During the second and third months of kindergarten, Aylin appeared to fluctuate between emotional withdrawal and joyful engagement. While she often wandered aimlessly through the classroom or garden in search of something to do, she also showed enthusiasm and excitement during structured activities that encouraged exploration. Activities prepared by Mrs. Burcu—who acted as a supportive and guiding presence—seemed to alleviate Aylin’s distress and foster more positive emotions.

Aylin’s social behavior also evolved during this period. At times, she interacted more openly with her peers and displayed confidence, happiness, and curiosity. Yet, these positive emotions were not always stable. Especially when her social initiatives were unsuccessful, frustration resurfaced. Moments of joyful self-expression, such as dancing in front of the mirror, alternated with signs of sadness and emotional strain.

A major transition occurred during the third month: Aylin’s class was merged with other five-year-old groups. This sudden structural change disrupted her adaptation process. She was observed wandering around the new classroom for extended periods, seemingly unable to find her place. Although she made some attempts to connect with her new peers, these efforts were inconsistent and often short-lived. Her repetitive movement patterns and withdrawn demeanor suggested that she was once again facing a difficult adjustment phase, marked by emotional stress and potentially compulsive-like coping behaviors.

### The fourth and the fifth month of the observation period

#### “I am growing up”

By the twelfth week, physical and behavioral shifts in Aylin began to signal a new developmental phase. One morning, the observer briefly failed to recognize her: Aylin was no longer wearing her glasses and had removed her shoes. These subtle changes were interpreted as signs of growing comfort and embodiment in the classroom, possibly tied to her immersion in imaginative play (see Supplementary Excerpt 14).

In a pretend play episode, Aylin passively took on the role of a “baby” while Duygu played the “mother.” Her lack of visible engagement suggested discomfort with the passive role. Soon after, she moved into the pretend kitchen and actively joined in the cooking play, a shift interpreted as her preference for adult-like, competent roles (see Supplementary Excerpts 15, 16).

Later that day, Aylin excitedly shared with her teacher that her first milk tooth had fallen out, beaming with pride. This small biological change seemed to anchor her emerging sense of identity. She laughed freely, danced, and sought to share this moment with peers, especially three boys—Hasan, Ege, and Salih—marking her first self-initiated interactions with male classmates (Supplementary Excerpts 17–20).

Her behavior grew more confident and expressive. She began to engage boys and girls alike, showing openness, initiative, and emotional fluidity. Her barefoot ballet in the center of the classroom—performed joyfully and without hesitation—became a symbolic expression of self-confidence and transformation. She no longer needed her glasses or shoes; her body, movement, and gaze communicated belonging and agency (see Supplementary Excerpts 21–22).

This phase of observation revealed a significant shift: Aylin no longer saw herself as a passive participant but emerged as an expressive and socially connected “young girl.” Her transformation was not simply behavioral, but affective and symbolic—driven by newfound emotional security, creative play, and social resonance.

### Countertransference and the aestheticization of the observational object

In the later phases of the observation process, a striking shift occurs—not in the child, but in the observer. What begins as an attentive effort to trace Aylin’s emotional life through her actions transforms, gradually but unmistakably, into a projection of aesthetic fascination. Rather than maintaining a stance of neutral inquiry, the observer finds herself captivated by Aylin’s drawing, compelled to change position in the room in order to follow the unfolding image more closely (see Supplementary Excerpt 23). The observational gaze is displaced by a kind of aesthetic enchantment.

The drawing, described in unusually elaborate terms, is interpreted as a depiction of “day and night” in the same visual field—an interpretation not anchored in the child’s verbalizations or behavior, but in the observer’s own symbolic associations. This moment marks a temporary suspension of the Tavistock ethos of *not-knowing*: the observer does not stay with the uncertainty of the child’s internal experience but rushes to fill the gap with meaning. The child’s creative act becomes the canvas for the adult’s unconscious (see Supplementary Excerpt 24).

The observer’s language slips into the register of art criticism. Comparisons with peers’ “stick figures” elevate Aylin’s work to a rarefied level, and her act of integrating another child’s drawing is framed as the construction of a multi-layered collage. In these moments, Aylin is no longer the subject of a developmental study, but the object of the observer’s projected longings and ideals (see Supplementary Excerpt 25).

As this pattern unfolded, the observer began to experience a visceral sense of injustice on behalf of Aylin—feeling pained by the child’s social rejections and moments of isolation. It became increasingly clear, particularly through seminar discussions, that the observer had begun to relate to Aylin not only as a researcher but with echoes of her own childhood experience. A sense emerged that Aylin “deserved better”—the best, in fact—and that her perceived talents and struggles needed to be seen, affirmed, even protected. This emotional alignment, while deeply human, blurred the boundary between observation and identification.

Yet within the Tavistock model, such moments are not seen as errors, but as openings. Through reflective dialogue, the observer came to recognize that her heightened emotional responsiveness may have been shaped by unconscious transference dynamics, likely rooted in personal history. In becoming aware of the intensity of her projections, she gradually recovered her observational stance—not by suppressing her feelings, but by metabolizing them. What had begun as unexamined countertransference was, through supervision, transformed into insight.

This crescendo of emotional investment culminated when the observer, anticipating criticism for idealizing Aylin, brought a photograph of the child’s drawing to the seminar in an effort to substantiate its artistic merit. She described other children’s drawings as “monotonous” and “lacking concept” —a response that was later recognized as a defensive gesture, aimed at preserving a fragile, affectively charged identification. In retrospect, the observer acknowledged this moment not as failure, but as a mirror held up to her own internal world—a rare opportunity to encounter, name, and revise the unconscious meanings she had inscribed onto the child (see Supplementary Excerpt 26).

In this light, Aylin’s drawing becomes more than a moment of expression—it becomes a site of mutual revelation. It reveals the child’s inner vitality, yes, but it also exposes the observer’s longing to rescue, to elevate, perhaps even to be seen. As Bion reminds us, the observer must become a container for the emotional field, not its editor. And as this case illustrates, the task of observation is not simply to look at the child, but to learn what the child awakens in us.

### Important information for the readers

After the 13th observation, the observer took a two-week break due to the semester break. During the two-week holiday period, the observer travelled to another city and witnessed an extraordinary disaster that deeply shook Turkey and unfortunately experienced two devastating earthquakes that occurred one after the other. The observer, who was in the midst of this traumatic process, which was materially and morally devastating, could not return to the city where he carried out his observation activities for about 3 weeks. Due to this unfortunate event after the semester break, the continuity of the study could not be guaranteed for more than a month.

### Overview of observations: fourth and fifth months

In the fourth month of the observation period, Aylin continued to experience difficulty in integrating into peer relationships. For the first time, she was the target of provocation by a classmate, and despite repeated verbal attempts to set boundaries, she was unsuccessful in stopping the behavior. This incident highlighted the lack of relational attunement between the children and marked a new threshold of frustration and vulnerability for Aylin.

Simultaneously, subtle but significant physical changes were recorded. In the second week of the fourth month, Aylin came to school without her glasses—an event that, while seemingly minor, would later align symbolically with a broader developmental shift. Shortly thereafter, it was observed that her first baby tooth had fallen out. This biological milestone became a focal point for self-definition. Aylin was no longer content to play the role of the “baby”; instead, she moved into the symbolic domain of adulthood by engaging in role play that involved cooking, caretaking, and the embodied performance of growth.

What followed was a striking sequence: Aylin, who had previously interacted almost exclusively with female peers, approached three male classmates—Hasan, Ege, and Salih—to show them the gap where her baby tooth had been. This moment marked her first direct social engagement with boys and coincided with a visible increase in confidence, autonomy, and ease in the classroom.

Perhaps most tellingly, Aylin then initiated and performed a ballet sequence alone in the center of the classroom. This action appeared to represent a kind of declarative presence: she occupied space freely, gracefully, and without hesitation. A classmate attempted to join her, but the dynamic was no longer one of rejection or exclusion. Instead, Aylin’s role had shifted—she was now the one who set the rhythm, the one to be followed. The earlier theme of painful non-belonging was inverted; Aylin no longer waited to be included. She performed herself into being. These events, along with subsequent observations of her physical, relational, and expressive changes, are documented in the Supplementary Excerpts 22–26.

### Sixth, seventh and eighth observation month

#### The nightmare in the botanical garden

In the sixth month, a contextual shift occurred as Aylin was observed outside the kindergarten setting during a school trip to a botanical garden. This new environment revealed distinct patterns in her social behavior and emotional regulation.

Upon arrival, Aylin displayed early prosocial behavior by offering her pink hat to a peer lacking sun protection—a spontaneous act of empathy and generosity (see Supplementary Excerpt 27). This gesture suggested that Aylin was internalizing peer group norms and beginning to act with social agency. A later exchange, in which she readjusted the hat with care, reinforced a growing capacity for relational attunement. However, subtle tensions emerged when another peer responded with visible discomfort, illustrating how inclusion and social positioning remain fluid in early peer groups.

Later, the group breakfast became emotionally dysregulated. Several children expressed distress, some crying or refusing food, while teachers struggled to restore order. Amidst this turbulence, Aylin remained composed—smiling, eating calmly, and maintaining emotional balance (see Supplementary Excerpt 28). Her behavior stood in contrast to the collective anxiety, pointing to increased emotional resilience and a capacity for self-regulation beyond the classroom.

However, this stability was not uniform. During a structured task led by the German teacher—counting aloud in a circle—Aylin became withdrawn. She responded hesitantly, barely audibly, and eventually disengaged entirely (see Supplementary Excerpt 30). This contrast revealed her discomfort with teacher-centered, performance-oriented settings, especially those lacking emotional safety or play-based context.

Seminar discussions highlighted that the didactic nature of the activity, mismatched with the outdoor setting’s sensory and exploratory affordances, may have suppressed engagement. A more flexible, movement-based pedagogical approach might have supported both participation and enjoyment (see Supplementary Excerpt 31).

### Overview of observations: months 6–8

The observations conducted during the sixth, seventh, and eighth months of the study revealed increasingly complex and differentiated patterns in Aylin’s social functioning, affective states, and cognitive engagement. Within peer interactions, a notable hierarchical structure began to emerge. Children appeared to assign and accept roles that mirrored adult-like power dynamics—some asserting authority and others adopting positions of compliance. Aylin’s participation in these role-based games demonstrated both her sensitivity to peer hierarchies and her flexibility within them. At times, she deferred to dominant figures such as Derin; at other times, she enacted roles of agency and strength—such as the “hunter” in a play scenario—suggesting a nuanced internalization of both submissive and assertive positions within the group’s imagined social order.

A particularly salient episode occurred when Aylin was brought to the kindergarten by both her mother and father for the first time. The emotional significance of this moment was palpable. Having been previously accompanied only by her father, the presence of her mother evoked intense joy and excitement in Aylin, which manifested in small but telling disruptions to her usual routine: putting her shoes on the wrong feet, forgetting to retrieve her school bag, and repeatedly kissing her mother. These behaviors suggest that Aylin was emotionally overwhelmed by her mother’s presence, which appeared to momentarily disorganize her but simultaneously elevated her affective state to a level of enthusiastic exuberance.

Importantly, this emotional uplift translated into increased cognitive and behavioral performance in the classroom. Aylin displayed rapid task completion, sustained concentration, and a willingness to support peers after finishing her own activities. This episode supports the hypothesis that strong maternal affect may serve as a short-term emotional catalyst, enabling children to access higher levels of cognitive and social functioning.

The final observation of the sixth month highlighted a potential turning point in Aylin’s peer relationships. A sustained and reciprocal interaction with Nil—characterized by extended verbal engagement and collaborative drawing—suggests the early formation of a friendship based on mutual interest and emotional resonance. Their effort to create matching artwork could be interpreted as a symbolic attempt to construct a shared imaginative world, reflecting their emerging emotional synchrony and the desire for dyadic belonging.

By the eighth month, observational data indicated a marked shift toward greater extroversion, verbal fluency, and socio-emotional ease. Aylin was increasingly able to articulate her thoughts, assert her presence in peer interactions, and demonstrate pleasure in shared experiences—particularly when a sense of group cohesion and inclusion was achieved. The contrast between her responses to structured, didactic instruction and free play further underscored the importance of autonomy and environmental fit in fostering her engagement. Whereas teacher-directed tasks elicited passivity and disengagement, open-ended, outdoor activities evoked spontaneity, joy, and a deepening sense of agency. These patterns suggest that Aylin’s developmental trajectory is highly sensitive to contextual affordances—thriving in relationally rich, play-centered environments that allow for self-directed exploration and expressive freedom.

### Latest observation month

#### The ice cream parlor: emergent creativity in the space of adult withdrawal

The final observation introduces a compelling shift in classroom dynamics. Upon entering, teacher Sema takes a seat at her desk, mutters half-jokingly that she does not want to work, and begins cutting cardboard, seemingly disengaged from the children (see Supplementary Excerpt 32). While this brief moment might be interpreted as professional fatigue, it inadvertently opens a space for spontaneous, child-led play. Rather than descending into disorder, the room fills with creative momentum—suggesting that temporary adult passivity, in a secure environment, can catalyze autonomy and imaginative collaboration.

In this context, Aylin, Zeynep, and Duygu spontaneously organize a complex and joyful role-play game: an “ice cream parlor.” Without adult direction, they assign roles, transform Lego blocks into ice cream scoops, and simulate economic exchange. Aylin emerges not as an outsider—as in earlier observations—but as an active co-creator, giving instructions, solving problems (“We forgot plain vanilla!”), and leading with enthusiasm (see Supplementary Excerpt 33). Her confidence and joy signal a developmental milestone in both executive functioning and social belonging.

The scene unfolds with layered symbolic interactions. Aylin, cradling a toy unicorn-cat and instructing Duygu to bring food, shifts fluidly between nurturing gestures and assertive leadership. Her ability to move between roles suggests a growing emotional flexibility and comfort within group storytelling (see Supplementary Excerpt 33).

This play episode also marks a shift in Aylin’s self-regulation and communicative ease. She no longer hesitates or withdraws. Instead, she initiates interaction—“Let us help too,” she says to Zeynep—and displays verbal spontaneity, sustained attention, and embodied presence. Compared to earlier moments when she avoided eye contact or responded with monosyllables, Aylin now engages fluently, signaling increased emotional security and social confidence (see Supplementary Excerpt 34).

The seminar group later noted this shift, observing that the same child who once seemed emotionally constrained now appeared animated and socially attuned. When freed from didactic constraints, Aylin expressed herself fully—reinforcing the developmental importance of autonomy-supportive, play-centered environments (see Supplementary Excerpt 35).

A further illustration of this group dynamic appears when Zeynep jubilantly exclaims, “We are rich, Ayliiiiiiiin, look at this money!” and Aylin echoes back with laughter, grabbing Duygu’s arm: “Duyguuuu, we are richuuuukk!” Their synchronized shouting and high-fives reflect not only joy but shared ownership over the symbolic world they have created (see Supplementary Excerpt 36).

This moment encapsulates a prosocial orientation in early peer play: shared authorship, mutual celebration, and inclusion. Zeynep’s use of “we” distributes symbolic wealth across the group, dissolving hierarchies. In contrast to adult economic systems marked by possession and rank, the children’s imaginative economy is egalitarian and affectively rich—grounded in reciprocity, delight, and belonging.

#### Overview of the last month

In the final month of the observation period, two sessions were conducted as the school year drew to a close. The 25th observation is particularly notable for its portrayal of a spontaneous and richly imaginative play scenario that unfolded after the teacher expressed a reluctance to engage in structured activities. What might have initially appeared as pedagogical withdrawal inadvertently created a fertile space for autonomous peer interaction and creative exploration.

Aylin’s role in the imaginary ice-cream parlour game reveals a significant developmental shift. Whereas she had previously avoided verbal participation or responded with minimal speech, she now initiates contact with enthusiasm—joining the game with the words “Let us help too” and contributing confidently throughout the interaction. Her verbal engagement and emotional expressiveness underscore a growing sense of agency and social integration.

Perhaps most striking is the egalitarian spirit with which the children navigated the game. The Lego blocks, symbolizing ice cream and money, were not hoarded but joyfully shared, and success was celebrated collectively. This dynamic reflects a peer culture free from adult-like competition or ownership, emphasizing instead mutual enjoyment and shared accomplishment—foundational experiences for cooperative social life in childhood (see Supplementary Excerpts 33–36).

#### Teacher presence and affective climate

Although the study primarily focuses on the child’s self-regulation and peer interactions, teacher presence—whether engaged or absent—was also observed to influence Aylin’s emotional state. During the early weeks of adjustment, emotionally distant or procedural interactions (e.g., simply reminding to wash hands without emotional attunement; Supplementary Excerpt 4) appeared to amplify Aylin’s discomfort. In contrast, even brief but emotionally responsive teacher gestures, such as a personal remark about her growing teeth, prompted visible expressions of joy and social re-engagement. These moments suggest that teacher affect plays a quiet but potent role in shaping the emotional landscape of the classroom, especially for children navigating transitions (Supplementary Excerpt 17).

### Theoretical anchoring and interpretive implications

The following synthesis draws on developmental theory to interpret Aylin’s evolving emotional, social, and symbolic competencies across the observational timeline.

The initial phase of observation, spanning the first 4 weeks of Aylin’s kindergarten experience, revealed a constellation of behaviors indicative of emotional strain and socio-relational inhibition. Her visible difficulty with the morning separation routine—marked by tearfulness and passive resistance—may be interpreted as a typical manifestation of early separation anxiety, consistent with Bowlby’s (1969) conceptualization of attachment-related stress and Ainsworth’s (1978, 2015) observational findings on insecure attachment patterns. Particularly in contexts where the transition to school lacks a structured orientation process, such reactions are likely to intensify. Research has shown that children’s ability to cope with separation is closely related to the emotional stance of their primary caregivers; as Peleg et al. (2006) demonstrated, maternal separation anxiety and low differentiation of self are strongly associated with children’s adjustment difficulties in early educational settings. Within this framework, Aylin’s early distress can be viewed not solely as an individual adaptation problem but as potentially relational in nature.

During the second and third months, Aylin’s behavior oscillated between cautious engagement and emotional withdrawal, a pattern often described in the literature as characteristic of socially withdrawn children who demonstrate situational variability in peer-related contexts (Rubin et al., 2009). Her intermittent enthusiasm during structured, adult-facilitated activities and her spontaneous expressions of joy—such as dancing alone—suggest that her emotional capacities were intact, but that she struggled with consistency in peer-driven exchanges. According to Qashmer (2023), such fluctuations in emotional regulation among 4–6-year-old children often correlate with variability in peer relationships, particularly when children lack the emotional tools to navigate social failure. The abrupt classroom merger in the third month introduced a new layer of environmental instability, which appeared to intensify Aylin’s stress response and activate repetitive, aimless behaviors. These may be understood as self-regulatory strategies—albeit limited ones—employed in the face of heightened uncertainty. This period thus reflects a complex interplay between emerging emotional resilience and persistent vulnerability, shaped by both interpersonal dynamics and structural disruptions in the learning environment.

The fourth through eighth months of observation revealed a notable developmental progression in Aylin’s emotional self-regulation, symbolic play, and relational presence. Her transition from vulnerability to agency was marked not only by biological milestones—such as the loss of a baby tooth—but also by symbolic actions that reflected a redefinition of self. As she moved from peripheral observation to initiating social contact, her play began to center around caregiving, performance, and role-based enactments. These observations align with findings by Bredikyte and Brandisauskiene (2023), who emphasize that pretend play offers a structured arena for the development of self-regulation through socially shared symbolic activity. In Aylin’s case, the internalization of autonomy was visibly enacted through solitary yet declarative performances, such as her ballet sequence, where she set the rhythm rather than followed it.

Moreover, the emergence of flexible social positioning—alternating between assertiveness and deference within peer hierarchies—suggests an advancing capacity for emotional modulation and social cognition. This echoes the longitudinal findings of Lucas-Molina et al. (2020), which demonstrate the co-development of emotional regulation and emotional knowledge in preschool-aged children. Aylin’s evolving ability to set boundaries, tolerate frustration, and adjust her behavior in light of peer responses also resonates with Rubin et al.’s (2009) conceptualization of social withdrawal not as a fixed trait, but as a dynamic interplay between motivation, skill, and context.

Episodes of relational expansion, such as her first engagement with male peers and her sustained interaction with Nil, further illustrate a shift from reactive to proactive modes of social engagement. These moments were often preceded or accompanied by affective surges, particularly in response to emotionally salient events like being accompanied to school by both parents. The emotional intensity of such experiences appeared to temporarily disorganize Aylin’s behavioral routine but concurrently elevate her cognitive and social responsiveness—a pattern supported by Qashmer’s (2023) assertion that heightened emotional states in preschoolers can transiently enhance or impair peer relations depending on the child’s regulatory capacity.

Finally, Aylin’s increasing verbal fluency, task focus, and joyful participation in unstructured outdoor activities indicate a developmental profile attuned to relational and environmental affordances. As Halfon and Bulut (2019) argue, the growth of symbolic play is closely linked to improvements in affect regulation, particularly in children who have experienced early challenges in social adaptation. Aylin’s progression over these months underscores the interdependence of emotional, symbolic, and interpersonal competencies in the early childhood landscape.

The final month of observation marked a significant developmental consolidation for Aylin. In the absence of teacher-directed structure, a peer-led symbolic play scenario emerged, offering fertile ground for autonomous participation. Aylin’s verbal initiation—“Let us help too”—reflected a shift in relational agency: from passive observer to co-author of the interaction. Her emotional attunement, verbal fluency, and confident engagement suggested that previously fragmented competencies had begun to integrate.

What stood out was the egalitarian dynamic of the game. Symbolic resources were joyfully shared, and peer roles co-constructed without conflict—creating a collaborative microculture. Lillard et al. (2013) argue that pretend play is most developmentally potent when it supports the negotiation of shared meaning and affect. In this sense, Aylin’s sustained participation illustrated an emerging capacity to regulate emotion within a socially generative space.

As Bodrova and Leong (2024) suggest, moments of child-led play allow autonomy and intersubjectivity to unfold organically. In this context, Aylin was not merely adapting to an existing order but actively contributing to its creation—embodying a form of social authorship rooted in imaginative reciprocity.

### Findings from video recordings

To enrich and triangulate the written observation protocols, selected excerpts from approximately 550 pages of transcribed video material were analyzed. These video recordings provided access to subtle, embodied aspects of Aylin’s behavior—such as posture, gaze, and pacing—that often escape written field notes. Through this visual lens, the core themes identified in the observational data were both affirmed and further deepened.

Instead of presenting the full dataset, this section highlights selected vignettes chosen for their illustrative power. Each segment reflects broader interactional dynamics and marks pivotal moments in Aylin’s social and emotional development—her shifting peer relationships, her oscillation between initiative and hesitation, and her efforts to find belonging in the classroom.

While the ethnographic protocols offered a narrative arc of Aylin’s development, video data allowed for microanalytic insight into affective shifts and peer engagement. These visual materials served to answer key questions about how Aylin experienced emotional life in the kindergarten and how her emotion regulation evolved over time.

Video recordings were scheduled for 1 h every 2 weeks between October 2022 and June 2023. Despite some interruptions due to holidays and absences, 13 sessions were completed. Although only selected scenes are presented here, all video data consistently supported the themes that emerged from written protocols. From the earliest adaptation phases, Aylin exhibited a wide range of emotional experiences—from withdrawal and ambivalence to joyful self-expression and creative agency. These multimodal records proved to be a valuable triangulation tool.

Still, certain emotional subtleties—especially tone and atmosphere—were at times difficult to perceive due to environmental factors like sound quality and camera angles. These limitations did not undermine the thematic integrity but highlighted the complementary strengths of each method. While written protocols conveyed emotional nuance and context, the video footage revealed embodied interactions and micro-moments of regulation that might otherwise have gone unnoticed. The excerpt below offers a representative scene that captures Aylin’s quiet struggle between emotional expression and relational withdrawal.

### Representative excerpts from video analysis

In the first video segment, Aylin stands at the periphery of the classroom, gently swaying with her arms crossed, watching a group of peers engaged in dramatic play. Though she does not join in, her posture and sustained gaze suggest a tension between the desire to belong and a fear of rejection. Small bodily cues—such as the curling of her fingers and shifting stance—indicate an internal negotiation. This moment captures the invisible emotional labor of navigating social space in early childhood (see Supplementary Excerpt 38).

Throughout the scene, while most children engage freely, Aylin remains on the edge—both physically and relationally. She walks aimlessly and silently observes, making no effort to initiate interaction. Despite being present for 2 weeks, she seems socially hesitant, possibly reflecting early signs of inhibition or uncertainty about attachment within the new environment.

An important moment unfolds when Salih approaches and speaks to her. Aylin does not respond—neither verbally nor with body language. Her silence appears emotionally muted rather than dismissive, possibly signaling cautious self-protection. This non-response may reflect both a lack of resonance with Salih and a deeper ambivalence about connection.

Later, Aylin quietly tells Salih, “You did not want me.” This short sentence—her first verbal expression in the video—carries significant emotional weight. It hints at a prior, unrecorded experience of social rejection. Her soft accusation suggests remembered pain, reframing her earlier silence not as indifference but as a response to being hurt. Immediately afterward, she exits the classroom, underlining a protective retreat after a vulnerable disclosure (see Supplementary Excerpt 39).

This scene illustrates how meaning in early childhood often lies as much in silence as in speech. Aylin’s quiet sentence gives voice to an inner world previously hidden—revealing early relational dynamics of rupture, memory, and self-protection.

### Findings from semi-structured interviews with parents

The interviews with Aylin’s parents revealed that she was born on 16 June 2017 as a planned and eagerly anticipated child. Her older brother is 4 years her senior. During the years before kindergarten, her maternal grandparents—both retired professionals—provided extensive childcare support, allowing both parents to continue working without the need for outside help.

When asked whether he spent enough time with Aylin, the father responded “no,” citing the pandemic as a limiting factor. He reflected on missed opportunities for shared experiences, such as camping, though his answers lacked specificity (see Supplementary Excerpt 40). He also described Aylin as “unfortunate” due to the pandemic’s timing, which overlapped with a key developmental period after her second birthday.

Although the father perceived Aylin to be more emotionally attached to her mother, the interviewer noted that classroom observations suggested otherwise. The father acknowledged that he deliberately acted warmly during drop-offs to ease Aylin’s transition into kindergarten, aware of her emotional sensitivity (see Supplementary Excerpt 41). He explained that his gestures—hugging and kissing—were intentional, aimed at preventing Aylin from feeling abandoned.

The mother, a mathematics teacher, described a close bond with her own father and emphasized the caregiving role played by her parents during Aylin’s early years. When asked about Aylin’s relationship with her father, she initially described emotional distance, attributing it to Aylin’s temperament. However, she quickly added that her husband was affectionate with both children, giving somewhat inconsistent responses—possibly out of concern for how her statements would be interpreted.

Over time, both parents reported an improvement in their relationship with Aylin. The father described more meaningful conversations during morning drives to kindergarten, while the mother noted that better work-life balance in recent years allowed for more connection.

When asked about Aylin’s recurring stomachaches, the mother reported that symptoms began early but were difficult to interpret. Although Aylin rarely verbalized pain, she showed signs of malaise, prompting medical evaluations. Given a family history of Familial Mediterranean Fever (FMF), this possibility was explored but ruled out after testing. The mother ultimately speculated that Aylin’s symptoms might have a psychological component, especially related to stress. She expressed uncertainty, stating, “I think it is a bit psychological, as if it causes stress,” acknowledging the possibility that Aylin’s somatic symptoms reflected underlying emotional distress (see Supplementary Excerpt 42).

### Assessment of the focus child’s expressive language skills

The researcher conducted two Havas-5 interviews with Aylin, once in the first week of kindergarten and once in the last week of kindergarten. Aylin’s pre-test and post-test scores were compared with the mean scores of the Havas-5 in Turkish, and the scores obtained are shown in the table below:

**Table 1:** 

Subcategories	Average	Pre-test	Post-test
A. Task Performance	17,6	11	17
B. Communication	12	5	10
C. Vocabulary	10,7	12	14
D. Grammar	IV	II	III
E. Sentence Formation	IV	II	IV

### Pre-test—October 2022

The researcher took Aylin to the Havas-5 interview on 6 October 2022, the first week she started kindergarten. Aylin was 5 years and 3 months old at the time of the pretest. According to the scores, Aylin scored 11 points in the Task Performance category of the Havas-5 Language Level Diagnostic Tool. It was observed that Aylin’s score in this category was below the average for the Turkish language. When the transcription of the Havas-5 interview was analyzed, it was found that Aylin did not describe the pictures in detail and some scenes were described “not at all” or “vaguely”.

When another category, ‘Communicative competence’, was analyzed, it was found that Aylin scored 5 points, which was below average. In this section it was noted that Aylin could not take the initiative in conversation and often needed instructions, and that her speech was always slow and slurred and therefore not fluent. Aylin did not speak for 25 s at the beginning of the interview and then answered the interviewer’s two questions with a single word.

In the vocabulary category, all the verbs used by Aylin were counted one by one. Compared to the average score of Havas-5 in Turkish, Aylin’s score was found to be 12 and it was found that she obtained a vocabulary score above the average.

Although no reference value was determined in the category “Grammar - Verb Affixes,” it was observed that children in the first grade of primary school reached level IV. Aylin remained at level II in this category by using the past tense suffix -miş and the present tense suffix -yor. In order for children to reach the fourth level in this category, they are expected to be able to use the past tense suffix -di and the present simple suffix -ir.

Finally, although there were no mean scores in the Sentence Formation—Conjunction Use category, it was observed that the children generally reached Level IV in the Sentence Production category, especially at the beginning of Grade 1. Aylin was able to produce subordinate clauses using simple conjunctions and infinitives, but she was not able to connect sentences using suffixes and she was not able to construct direct transfer sentences. Therefore, Aylin remained at level II in the related category.

### Final test—June 2023

The researcher took Aylin to the Havas-5 interview on 7 June 2023, during her last week in kindergarten. Aylin was 5 years and 11 months old at the time of the final test. According to the scores, Aylin scored 17 points in the Task Fulfilment category of the Havas-5 Language Level Diagnostic Tool. It was observed that Aylin’s score in this category was below the average for the Turkish language, but her score increased significantly compared to the pre-test. When the transcription of the Havas-5 interview was analyzed, it was found that Aylin did not describe only the first picture in detail, some of the other pictures were complete and some were vague.

In another category, ‘communicative competence’, Aylin scored 10 points, which is below average, but she doubled her score compared to the pre-test. The most striking difference in this section was that Aylin sometimes took the initiative in speaking and spoke spontaneously without needing to be instructed.

In the ‘Vocabulary’ category, the number of verbs used by Aylin was 14, which was above average. In the category “Grammar - Verb Affixes,” Aylin reached level III by using the suffix “-yormuş” compared to her first results. Finally, in the category “Sentence Formation—Use of Conjunctions,” Aylin was at the same level as the children who started the first grade—Level IV.

In order to get an indication of Aylin’s expressive language skills, the Havas-5 Language Diagnostic Tool was administered to the focus child Aylin twice, once at the beginning and once at the end of kindergarten. The results showed that Aylin scored well below average on the pre-tests, but scored close to average on the post-tests. Although it would not be correct to make definitive judgements about Aylin’s expressive language performance, it is thought that the results provide clues to clarify some issues. In this context, it has been observed that expressive language performance is related to emotion regulation processes. During the first weeks and even the first months of kindergarten, Aylin showed an abstinent, anxious, passive and withdrawn behavior and her expressive language performance was below average in direct proportion to her emotion regulation processes. During the Havas-5 interviews it was observed that Aylin did not take the initiative, remained silent for seconds and gave one-word answers. In this respect, before linking Aylin’s language performance to her inadequate language skills, it should be considered that there may be some underlying emotional reasons. It should be taken into consideration that Aylin may have felt anxious and stressed during the interview with the interviewer in this kindergarten environment, which was probably the first time she had been in it. Similarly, assuming that Aylin had adapted well to the kindergarten at the time of the post-test and had taken the presence of the interviewer for granted, Aylin’s performance can be associated with her feeling more comfortable and free, and displaying a more extroverted and independent attitude. In this context, it can be inferred that Aylin’s emotional processes are related to her expressive language performance. These findings suggest that expressive language development in early childhood may not be fully understood without considering the emotional conditions that scaffold or inhibit verbal risk-taking and interpersonal engagement. Given the relationship between emotional regulation and language development, the findings highlight the importance of considering emotional well-being in language intervention strategies for preschool children.

### Responses to the research questions

1 What kinds of social interaction experiences did the focus child have with peers and teachers during the kindergarten year?

Throughout the kindergarten year, the focus child, Aylin, was observed engaging in a range of social interaction experiences that evolved significantly over time. Initially, Aylin exhibited withdrawn behavior, hesitated to initiate contact with peers, and often positioned herself on the periphery of group activities. Her interactions were marked by cautious observation and tentative gestures toward inclusion, which were sometimes met with subtle rejection or indifference. However, as the months progressed, Aylin began to demonstrate increased social agency. She made repeated efforts to initiate play, offered objects as symbolic gestures of connection (e.g., Lego figures, pretend food), and eventually participated in complex group narratives such as the “ice cream parlor” scenario. Her evolving ability to both observe and co-create social meaning within peer dynamics reflects a deepening engagement with the emotional and relational fabric of the classroom. Interaction with teachers remained largely instrumental; however, spontaneous moments of connection—particularly with more attuned educators—provided emotional scaffolding during transitions and moments of vulnerability.

2 What developmental changes occurred in the child’s emotional life, and how did she experience processes of emotion regulation?

Aylin’s emotional life evolved from early expressions of vulnerability and ambivalence—such as silent crying during morning separations—to increasingly nuanced and regulated affective responses. In the early stages, she often appeared overwhelmed by transitions, withdrew during emotionally charged group settings, and demonstrated difficulty navigating peer rejection. Over time, she began to exhibit signs of growing emotional regulation: taking initiative, recovering from setbacks, and showing empathy toward peers. Her responses became less reactive and more adaptive, especially in emotionally safe, play-rich environments. Notably, her verbal acknowledgment of exclusion (“You did not want me”) during one video segment, followed by self-initiated withdrawal, suggests an emerging capacity to articulate and manage relational disappointment. Such moments mark a developmental progression from pre-verbalized emotional responses to symbolic and socially aware expression.

3 What types of changes and progress were observed in the expressive language skills of the focus child during the observation process?

Aylin’s expressive language skills showed marked improvement over the course of the observation period, as evidenced by both structured assessment (HAVAS-5) and naturalistic observations. Initially characterized by short, hesitant responses and limited grammatical complexity, her verbal output expanded significantly by the end of the school year. She began to use more varied vocabulary, complex sentence structures, and self-directed speech. These changes paralleled her emotional and social development; greater emotional comfort and peer engagement appeared to unlock her linguistic expressiveness. Particularly in imaginary play contexts, Aylin demonstrated narrative fluency, turn-taking in dialogue, and the ability to use language as a tool for co-creating shared meaning. Her improved grammar and sentence formulation—reflected in both test results and daily classroom discourse—support the close interplay between emotional security and language development in early childhood.

4 What insights can be gained about the focus child’s daily life outside of kindergarten, and how do her parents describe their thoughts and feelings about her?

Semi-structured interviews with Aylin’s parents revealed a complex backdrop to her kindergarten experience. Born as a planned and highly anticipated child, Aylin benefited from the consistent caregiving of maternal grandparents and shared a household with an older sibling. However, the COVID-19 pandemic significantly disrupted her early socialization, limiting peer interaction and physical play for nearly a year during a formative period. The father described limited involvement due to work demands but acknowledged a deliberate effort to convey affection during goodbyes—moments that were emotionally rich in the observation records. The mother, while somewhat inconsistent in her descriptions—possibly due to the recording context—expressed concerns about Aylin’s emotional sensitivity, frequent stomachaches, and a potential psychosomatic origin for some of her physical complaints. Both parents acknowledged emotional distance in the early years, which they felt had improved recently. Collectively, these insights provide a fuller understanding of Aylin’s emotional life and highlight the connection between her home environment, early attachment patterns, and adaptive behaviors within the kindergarten setting.

## Discussion and recommendations

This case study provides valuable insights into early socio-emotional development, focusing on emotion regulation and the link between affective security and expressive language in a five-year-old’s kindergarten transition. Using the Tavistock Child Observation Model, videography, and parental interviews allowed for an in-depth view of the child’s relational and emotional journey. Aylin’s early months in kindergarten were marked by significant emotional turbulence. Her repeated distress during separations from her father and minimal engagement in classroom activities signaled attachment-related vulnerability and difficulty adjusting to the abrupt transition from home. The lack of a structured adaptation process—such as the Berlin or Munich settling-in models—appeared to heighten this disruption, echoing findings on the emotional costs of unsupported early separations (Battaglia et al., 2015; Czada, 2012; Frisch, 2012; Xiao, 2015).

Initially, the classroom failed to offer emotional containment. Teachers were present but emotionally distant, limiting Aylin’s ability to experience the environment as secure. Gradually, she developed strategies for social engagement, shifting from passive observation to active participation in imaginative peer play. These interactions—especially the “ice cream parlor” episode—illustrate how spontaneous, child-led play fosters emotional regulation and connection when supported by attuned but unobtrusive adults. Building on Vygotsky’s (1978) and Winnicott’s (1991) foundational theories, recent studies (e.g., Galyer and Evans, 2001; Veiga et al., 2022) underscore the role of symbolic play in supporting emotional integration and social development in early childhood.

Aylin’s capacity for emotion regulation developed alongside her increasing social integration. Early signs of withdrawal—such as silent crying—gradually shifted toward expressive behaviors, including verbalizing exclusion and engaging peers in play. This trajectory illustrates that emotion regulation is co-constructed in relational contexts, rather than purely internal. These observations align with research emphasizing peer interaction as a critical scaffold for both emotional and social competencies (Denham et al., 2007; Eisenberg et al., 2010; Blair and Raver, 2015; Kraiss et al., 2020; Kwon et al., 2024; Hladik et al., 2024).

Similarly, changes in Aylin’s expressive language were closely tied to emotional security. Her low HAVAS-5 pre-test scores likely reflected early stress during the transition period, while her improved post-test results paralleled observed increases in fluency, grammar, and narrative ability. These findings reinforce the notion that emotional well-being enhances verbal development and are consistent with studies linking expressive language to affective and relational contexts (Polzer, 2012; Datler et al., 2014a; Wong et al., 2023; Alamos et al., 2022; 2025).

Parental interviews added depth to the findings by revealing inconsistencies in how Aylin’s behavior was interpreted at home. While both parents described her as emotionally sensitive, they often compared her to her older sibling rather than attending to her individual developmental trajectory. The COVID-19 pandemic further limited Aylin’s early opportunities for peer interaction and autonomy, likely contributing to her initial adjustment difficulties (Watts and Pattnaik, 2023).

A key insight was the mismatch between parental perceptions and observed behaviors. Described at home as “easy” or “low-maintenance,” Aylin was in fact grappling with emotional stress and a need for relational containment. This highlights the importance of integrating multiple methods in early childhood research and the unique value of direct observation.

### Recommendations

1 **Structured Adaptation Models:** The lack of a settling-in process appeared to hinder Aylin’s adjustment. Models like the Berlin or Munich approach can offer vital emotional scaffolding for children entering school settings.
2 **Emotionally Responsive Pedagogy:** Emotionally safe, play-centered classrooms foster both affective and cognitive growth. Teachers should be trained to attune to children’s emotional cues, particularly during transitions.
3 **Peer Interaction as a Learning Tool:** Aylin’s most significant developmental gains occurred during peer-led play. Unstructured social interaction should be embedded into early education practices.
4 **Context-Sensitive Language Assessment:** The emotional state during testing impacts language outcomes. Standardized assessments like HAVAS-5 should be paired with observational methods to better capture children’s communicative abilities.
5 **More Case Study Research:** This study underscores the value of detailed case studies in uncovering developmental processes. As Fatke (1995) emphasizes, linking multiple case studies enhances the generalizability of individual findings.

Through an integrated methodology combining observation, videography, and parental insight, this study traced Aylin’s journey from silent withdrawal to confident engagement. Her case highlights the need for early childhood environments that support both emotional resilience and linguistic growth.

## Conclusion

The case study followed Aylin throughout her first year of kindergarten while documenting her emotional, social and linguistic development using naturalistic observation alongside videography and standardized assessments complemented by thorough parental interviews. The unfolding scenario presented a complex developmental journey marked by the influences of relationships and transitions alongside significant emotional connections.

During the initial days of school Aylin remained motionless at the classroom perimeter exhibiting quiet behavior and internal withdrawal. Her behavior showed uncertainty and reluctance to interact or speak which went beyond typical shyness as she faced the unfamiliar emotional challenges of school life for the first time. The transition from home’s stable patterns to the unpredictable nature of group interactions imprinted itself onto her early behavior because she lacked a gradual introduction to the new environment.

Over time, however, subtle shifts emerged. In shared play, in the small rituals of classroom life, Aylin began to extend herself—first tentatively, then with increasing fluency. Her development unfolded not through dramatic leaps but through accumulations of relational moments: a shared laugh, an exchanged glance, a turn taken in a game. These interactions signaled more than social adaptation; they marked emotional risk-taking and growing self-trust.

Aylin’s linguistic progress mirrored this emotional journey. In the early months, her speech was sparse, marked by hesitation. But as she began to feel more anchored in the social fabric of the classroom, her verbal expression grew more confident, her vocabulary richer, her sentences more fluid. Language, for Aylin, became not just a cognitive achievement but a sign of felt safety and connection.

This study suggests that symbolic play, emotional regulation, and language development are not isolated domains but mutually informing processes. Aylin’s growth was most visible in moments when adult presence stepped back just enough to let her imagination lead—moments where she could shape meaning in her own time and language. The “ice cream parlor” scene, for example, was more than play; it was a declaration of social belonging and expressive agency.

Such developmental shifts often go unnoticed without sustained, longitudinal observation. The small transitions—between stillness and motion, silence and speech, solitude and connection—speak volumes about a child’s inner life. Aylin’s story reminds us that early adaptation is not linear. It ebbs and flows, responding to the emotional atmosphere of the classroom, the reliability of adult containment, and the availability of symbolic space.

For educators and researchers alike, Aylin’s year in kindergarten is a quiet but powerful testimony. It invites us to attend more carefully to the unspectacular, the fleeting, the easily missed signs of growth—and to trust that in these moments, development is indeed unfolding.

## Data Availability

The original contributions presented in the study are included in the article/Supplementary material, further inquiries can be directed to the corresponding author/s.
