# Supplementary material for: A qualitative research on emotion regulation processes and expressive language skills in kindergarten: a case study
Source: Front Psychol. 2025 Jun 23;16:1611554. doi: 10.3389/fpsyg.2025.1611554 (PMC12230095; doi:10.3389/fpsyg.2025.1611554)
Supplement: Supplementary file 1 [file Table_1.DOCX]

**Supplementary Material: Tavistock Observations**

This document includes selected observational notes recorded during a yearlong Tavistock-based case study of a 5-year-old child in a kindergarten setting. Each entry reflects real-time observation and reflective interpretation of the child’s emotional expression and group interactions.

**Explanation of Structure**

This supplementary material contains selected observational excerpts that support and elaborate the findings presented in the main manuscript. The document is organized chronologically, covering key moments from the longitudinal observation period of the focus child, Aylin, during her first year of kindergarten.

Each entry is labeled as **“Supplementary Excerpt [number]”** and corresponds to a specific moment or episode referenced in the main text. These excerpts offer detailed, naturalistic descriptions of Aylin’s behaviors, verbal expressions, peer interactions, and emotional states. Where relevant, the document includes interpretive comments and seminar reflections based on group discussions conducted during post-observation meetings.

The excerpts are grouped by developmental period (e.g., early adjustment phase, mid-year transitions, late-stage symbolic play), allowing the reader to trace Aylin’s emotional, social, and linguistic progression over time. Several excerpts also include verbatim quotes or transcribed dialogue that illustrate key themes such as emotion regulation, imaginative play, and relational dynamics.

This material is intended to provide transparency regarding data collection and analysis, and to illustrate the methodological grounding of the case study. All personal names have been anonymized. The excerpts were selected for their illustrative value and do not represent the full corpus of observations.

**Note on Numbering:**

Each excerpt is followed by a reference (e.g., Observation 2/1), where the first number indicates the observation number in the original field notes, and the second refers to the page or entry number from which the excerpt was selected.

**Supplementary** **Excerpt 1**

A child enters the nursery gate with his mother. As the child sobbed, I heard his cries: "Mummy, don't leave me! Mummy, don't leave me!" The mother, with her other baby in her arms, waits for her crying child to put on her shoes. Meanwhile, I hear her saying to the security guard at the door, "Ayyy, he was sick for a week, we had to wind him back, he was used to it". Then she turns to her child and says: "Enough is enough, everyone is going to school, stop crying, I'm so bored of going through this all the time". The child reaches out her hands to her mother and says with sobs, "Mummy, please take me with you, Mummy, please" (Observation 2/1).

**Supplementary Excerpt 2**

Then another parent arrives to hand over her baby. She hands her baby to the guard and gives him a light kiss, smelling his hands with her eyes closed. The baby on the guard's lap looks at his mother. The smiling face of the baby is replaced by a frown, a wrinkled mouth and a reddened face as the security guard walks away - step by step away from the mother (Observation 2/2).

**Supplementary Excerpt 3**

Just as I am going down the stairs, I notice Aylin coming up the stairs. Aylin is rubbing her eyes and inhaling to keep her reddened nose from running. I realised she was crying silently and wiped away her tears, trying not to let anyone notice. She continues to walk up the stairs with slow steps, following them without looking back. I stay on the stairs and watch Aylin for a while and then start walking behind her (Observation 2/2).

**Supplementary Excerpt 4**

After Aylin enters the classroom, Mrs. Burcu *reminds her to wash her hands and* says "Hands!". Aylin comes out of the classroom again and after bending down and lingering in front of the door for a while, she goes back inside. She does not go to wash her hands*.* She waits at the classroom door for a while. In the meantime, Aylin somehow stops crying, looks expressionlessly at her friends sitting on the floor and slowly moves towards the cushions (Observation 2/2).

**Supplementary Excerpt 5**

As I took my seat on the bench, I heard the voice of a three-year-old boy screaming and crying at the entrance to the nursery. His mother tried to calm her son, who had wrapped his arms and legs around her like an octopus, by saying: "But don't do that, my love... I'm coming... I'm coming... don't cry anymore...". The child's crying did not stop, and this time his mother said, "If you continue to cry, I won't come to get you in the evening. I won't pick you up". The child's sobs continue to increase. I was afraid he was going to have an attack of rigor mortis. The teacher on duty, who had been greeting the children at the door, walked briskly down the corridor and came to the mother and child. "Come here, come here, come on, don't cry, my dear," she said, trying to separate the child, whose arms and legs were wrapped tightly around his mother's body, and taking him in her arms with difficulty. The child's voice is now so loud that it seems to penetrate the walls. As the teacher walked past me with the child in her arms, I felt a sudden ringing in my right ear as the child screamed and cried as if her tonsils were bursting (Observation 4/1).

**Supplementary Excerpt 6**

Aylin puts the palms of both her hands together and pretends to pick something up from the table, taking small, quick steps and moving carefully towards the teapot on the cupboard. She holds it in her palms as if it were full of liquid. She lifts the lid of the teapot and pours the imaginary liquid in her palms into the teapot. She closes the lid and continues to fill the remaining cups (Observation 1/4).

**Supplementary Excerpt 7**

Based on the observation section where the observer describes Aylin as carrying an imaginary liquid, it can be suggested that what she is carrying may well be tea and that she is brewing a cup of tea. Aylin is probably making this tea to serve to her friends (Seminar 1).

**Supplementary Excerpt 8**

Aylin looks at the coasters in the pink toy basket. She takes three of them and chooses three of the dozens of cups in front of her and places them on the coasters. Then she begins to place these three glasses on his left arm, as there is no tray. As she places the last one on his arm, he drops one of the toys on the cupboard on the floor. When she bends down to pick up the toy, the glasses in her arms also fall to the floor. Aylin puts them all back on the cupboard and starts arranging them again (Observation 1/5).

**Supplementary Excerpt 9**

Aylin plays with Lego by herself for a while. It can be seen that she is trying to make a figure with a great deal of care and attention. She is trying to get three identical figures. After watching what her friends were doing for a while, Aylin picked up the Legos and went over to Nil and Esra who were sitting on the floor in the middle of the classroom playing with Legos. She sits down quietly next to them and asks with some hesitation, "Can I play with you too? Esra, who is concentrating on her game with Nil, looks at the Legos in front of her and replies, "You can't". Aylin presses her lips together, curls them inwards, stands up gently, picks up her Legos and puts them back on the cupboard. I feel that she is upset (Observation. 9/3).

**Supplementary Excerpt 10**

Aylin puts her Lego back on the cupboard and starts to play. I can see her talking to herself, but I cannot hear what she is saying. When she says something, she confidently and skilfully disassembles, inserts, removes, reassembles and finally transforms the three figures she had previously made into two identical figures. He puts them side by side and watches them for a while (Observation 9/3f).

**Supplementary Excerpt 11**

Aylin turns around and begins to watch her friends. For about 7-8 minutes she watches them intently. Someone is baking a cake, someone is making party tickets, someone is making a sofa out of jigsaw puzzles. After watching for a long time, Aylin picks up her Legos and carries them to the cupboard. I can tell she wants to be a little closer to her friends who are playing. On the other side of the cupboard, Duygu holds the puzzles in her hand like a tray and asks, "Who wants quince cake?" The children, including Aylin, answer "I do" in unison. After offering some cake to a few people next to her, Duygu puts the puzzles down as if she had remembered something and starts looking for another toy. Aylin, who did not get any quince cake, starts looking at her Legos again (Observation 9/3).

**Supplementary Excerpt 12**

While Aylin is playing with her Lego, Duygu starts to serve everyone with another puzzle, which she holds in her hand like a tray. She cheerfully approaches Aylin and asks, "Do you want some?" Without taking her eyes off her Lego, Aylin shakes her head from side to side and replies, "No, I don't", as if she wants Duygu to leave her side as soon as possible (Observation 9/3).

**Supplementary Excerpt 13**

I see Aylin looking at Duygu. Meanwhile, Duygu is showing the red Lego piece to her friends and says: "These are the tickets. Then Aylin quickly looks at the table. After looking around for a while, she goes to the Lego figure she had put aside, takes out a piece of red Lego and starts walking towards Duygu. When she gets to Duygu, she smiles and holds out the red piece of Lego and I hear her say, "I have a ticket too. Duygu replies, "You were supposed to get it from me, but never mind." Duygu looks at Aylin for a moment with a friendly smile and starts to check the party supplies again. The smile slowly fades from Aylin's face. Holding the red Lego piece in her hand, she looks around with blank eyes. I am almost certain that her heart is in pieces (Observation 9/4).

**Supplementary Excerpt 14**

I find it difficult to distinguish Aylin from the other children because I notice that she is not wearing her glasses today. Then I noticed that she did not have her shoes on. I think I have never seen her without shoes in the classroom before. After a while I see that her shoes are lying on the floor nearby and I think that she probably took them off because of the scenario of the game they were playing (Observation 12/1).

**Supplementary Excerpt 15**

Duygu holds Aylin's hand and says, "Baby, you are going to bed now". Then she takes Aylin's arm and puts her on the sofa. Meanwhile, she strokes Aylin's head like a baby and tries to put her to sleep by closing her eyes with her hands. There is no expression of pleasure on Aylin's face (observation 12/2).

**Supplementary Excerpt 16**

Meanwhile, Aylin is bored of playing the baby and gets up from her seat. Together with Duygu, they move to the kitchen area of the Dramatic Play Centre. I see them starting to cook with the plastic plates and pots on the toy stove. Using a small toy spoon, they cook the food they have made from the plastic vegetables in the pots and plates (Observation 12/2).

**Supplementary Excerpt 17**

When I could finally concentrate on Aylin, I saw that teacher Sema was standing next to her. I see her pointing at something by putting her index finger to her mouth. Teacher Sema smiles and says: "Aaayyyy you are growing up nowk Ayliiin congratulations, baby teeth are coming out, give me five". I can't see Aylin's expression because her back is turned, but after high-fiving teacher Sema with her hand, she turns and starts walking towards the class and I see the big smile on her face. She even starts jumping up and down on her seat and walks towards her friends with a smile I have rarely seen, which I am sure is joyful, until her eyes remain small in their sockets and her mouth is wide open on both sides (Observation 12/3).

**Supplementary Excerpt 18**

She approaches Hasan (a boy), with whom I'd never seen her speak before, and, restraining her enthusiasm, calmly touches Hasan's arm. "Hasan, Hasan, look," she says, pointing with her index finger to the gap in his extracted lower tooth (Observation 12/3).

**Supplementary Excerpt 19**

Aylin continues to smile and this time she jumps up and goes to Ege. I hear her say calmly, "Ege, look, my tooth came out. Ege just says, "I understand. Aylin leaves Ege's side and walks around the classroom again with great joy (Observation 12/3).

**Supplementary Excerpt 20**

She then approaches Salih and says "Saliiih, look", again showing the jaw gap (Observation 12/4).

**Supplementary Excerpt 21**

Aylin smiles in the middle of the class and starts to do ballet movements. Meanwhile, she notices Merve watching her and says "I'm going to do ballet" while looking at her. Merve quickly gets up from her seat and says "Me too". Again, I can't hear what Aylin is saying because of the noise in the classroom and because she is speaking in a low voice. Merve answers Aylin with a loud "Then let me be your pupil". I think Aylin must have told Merve that she doesn't know ballet. Immediately afterwards, Aylin starts doing ballet moves in the middle of the class and Merve imitates what she is doing. She stands on her tiptoes, turns around, jumps up by spreading her arms to the side and lifting one leg, and turns around again... (Observation 12/4).

**Supplementary Excerpt 22**

In the observation report, the observer thinks that Aylin might have taken off her shoes as part of a play scenario. However, when we look at the later parts of the observation report, the idea that Aylin might have taken off her shoes for the ballet performance she was going to perform comes to the fore. On the other hand, the fact that she came to the kindergarten without her glasses is a sign that Aylin was starting to feel more like a young girl (Seminar 12).

**Supplementary Excerpt 23**

Since I can't see Aylin's drawing from where I'm sitting, I get up and take a few more steps towards the table, lean against the wall and continue to watch. My eyes fall on Nil's paper. With a crayon in his hand, Nil begins to colour his paper with exactly the same colour, as if he were scribbling rapidly (Observation 13/4).

**Supplementary Excerpt 24**

Meanwhile, looking at Aylin's almost finished painting, I notice a detail. Surprised and in silence, I say to myself, "She is painting day and night in the same picture". I am impressed by the fact that he has painted the planets on a black background following the blue sky on a green background. The fact that he has combined day and night in the same picture makes me want to look at the other children's pictures again. When I see the stick men they have made, I think that this picture is different from theirs (Observation 13/5).

**Supplementary Excerpt 25**

It can be seen that the observer has started to transfer better and better, comparing Aylin's drawings with the drawings of other children. At the same time, it is noticeable that he underestimates the works of the others and glorifies Aylin's works; he treats the children's pictures like an "art critic". On the other hand, during the meeting, the observer realised that day and night were only his own interpretation and criticised himself that his perspective had become blurred at some point (Seminar 13).

**Supplementary Excerpt 26**

I see Aylin sitting on the floor, tapping her feet in rhythm. I feel that she is full of enthusiasm. On the other hand, I continue to watch carefully, wondering what will be added and developed to this work, which is no longer just a picture, but has taken on different dimensions. ... I also find it very creative that Aylin has cut and pasted Zeynep's picture as a colourful cotton candy, giving the planet book a collage dimension. When I look at the final result of Aylin's work, I think that Zeynep might have added the picture she made as a cotton candy to this work, perhaps thinking that it was a human being. I have the idea that in this work, where day and night are together, there could of course be a colourful human being (Observation 13/6).

**Supplementary Excerpt 27**

Fatma says, "I don't have a hat," in a sad and worried voice. Aylin rushes to her bag on the bench and takes out a pink hat. She hands the hat to Fatma and says with a smile, "You can wear this". Fatma smiles and puts the hat on her head (Observation 22/2).

**Supplementary Excerpt 28**

Fatma comes up to Aylin and gives her the hat, saying "Here, this is not right for my head" and smiling shyly. Aylin takes the hat and puts it back on Fatma's head, pulling it from left to right and smiling and saying "It's done". Fatma smiles back, touches the hat on her head and goes back to her seat. Fatma and Duygu sit next to each other and Aylin sits opposite them. When Fatma takes her seat, I see Duygu frowning and looking at Aylin (observation 22/2).

**Supplementery Excerpt 29**

While the teachers were distributing the breakfast sandwiches, most of the children did not want to eat and refused the sandwiches offered by the teacher. Meanwhile I see that Esra is not with her friends sitting in a circle, she is standing under the tree and crying. The security guard was right next to Esra, hugging her and trying to calm her down. Just as I'm looking at Aylin, Fatma screams and jumps up from her seat, saying "Teacher, I'm scared!" and hugging teacher Burcu's arm. I realise that she is afraid of an insect. While all this is going on, Bilge starts to sob because he is afraid of insects. When I can divert my attention from the other children, I look back at Aylin. I see that she has eaten half of her sandwich and is looking around with a very pleasant smile on her face. Teacher Burcu is trying to calm Fatma, while teacher Sema is trying to calm Bilge. I can see from their faces that both teachers are tense. On the other side, the security guard is still looking after Esra. Teacher Hilal is removing the tomatoes that Duygu refuses to eat from her sandwich. Really, everyone seems tense and unhappy (Observation 22/2f.).

**Supplementery Excerpt 30**

After some children have counted, it is Aylin's turn. Aylin looks at Mrs Hilal without saying anything. Mrs Hilal tries to encourage Aylin to start counting by saying "One..." and waits. After a short pause, Aylin says "One" quietly and shyly. Then Mrs Hilal waits a little longer and says "Two...". Aylin repeats in a voice so low that it is almost inaudible. Mrs Hilal must have realised that Aylin did not want to count, so she started to count on her own without pausing. Aylin repeats only a few of the numbers, her voice getting lower and lower (Observation 22/4).

**Supplementery Excerpt 31**

It is noteworthy that the focus child, Aylin, was instructed by her German teacher to count numbers with different instructions and that the child spoke in a low voice and did not show any facial expressions to indicate that she was enjoying herself. Sitting the children in a circle is designed to keep them together rather than to herald a fun activity. In other words, instead of sitting the children in a circle and teaching them monotonous counting based on repetition in the form of "Eins...Zwei...", they might have learned by playing with a ball in a game. Teaching German to children in the Botanical Garden removes all favourable atmosphere and conditions (Seminar 22).

**Supplementery Excerpt 32**

Mrs Sema goes to a table and starts cutting cardboard. I hear her say, "I don't want to work today." When I looked back at her, I realised she was talking to me. Then she says, "I wonder if I have such a chance, no" and laughs a little (Observation 25/3).

**Supplementery Excerpt 33**

Zeynep gives Aylin and Duygu the colourful Lego blocks she has stacked on top of each other and says: "This is lemon, this is cocoa, this is pistachio, this is strawberry" and shows them one by one. Aylin suddenly stands up and says, "Aaaaaayyyy, we forgot plain vanilla ice cream! We won't be able to play without it! Duyguuu go and get some white Lego!" she shouts enthusiastically. Duygu gets up to do as Aylin says and goes to the block centre to look for white Legos. Meanwhile, the customers start to arrive again and line up in front of the counter. Zeynep asks them one by one what kind of ice cream they want and how many scoops they want. Aylin takes out the coloured Lego blocks that match the customers' requests and hands them to Zeynep. Zeynep takes the money for the ice creams from the Lego pieces and puts them in the small space between her and Aylin. ... Duygu comes back to the counter with white Lego pieces and hands them to Aylin. Aylin says: "Here are the plain ice-creams" and puts the Legos on the ice-cream stand. Meanwhile, the number of children waiting in the queue increases and Zeynep quickly takes orders while Duygu and Aylin prepare the ice creams. Aylin smiles all the time. She looks very happy. This is the first time I have seen her play such a planned game (Observation 25/4).

**Supplementery Excerpt 34**

Aylin looks at Zeynep with a smile and says "Let's help too". Zeynep says, "Ok, now it's closed, the ice creams are being prepared, look at this" and takes a toy in the shape of a unicorn cat out from under one of the boxes standing next to each other. Aylin immediately grabs the toy, puts it on her left arm and starts to shake her arm from side to side, as if she was trying to put a little baby to sleep. Then she smiles and says "Duyguu, bring some food" (Observation 25/3).

**Supplementery Excerpt 35**

It can be seen that the girl who two weeks ago did not seem willing and enthusiastic to answer the questions asked by the teacher, who was bored, who was playing with her claw buckle, who was pulling her T-shirt up to her feet, who did not want to talk, changed into a more extroverted, excited girl who talked more (without hesitation) when she was left free and freely part of a game (Seminar 25).

**Supplementery Excerpt 36**

Zeynep looks at the Legos piled up next to her and excitedly nudges Aylin and says: "We are rich Ayliiiiiiiiiin look at this money". Aylin's smile covers her whole face and she grabs Duygu's arm and shakes her and says "Duyguuuu we are richuuuukk look at this" and shows the Legos. They all shout with joy, raise their hands in the air and high-five each other (Observation 25/4).

**Supplementery Excerpt 37**

In the game, the aim is not to be the boss or to buy/sell all the ice-creams requested, they are not surrounded by ambitions like adults. The relevant section shows that children do not seek power like adults (Seminar 25).

**Supplementery Excerpt 38**


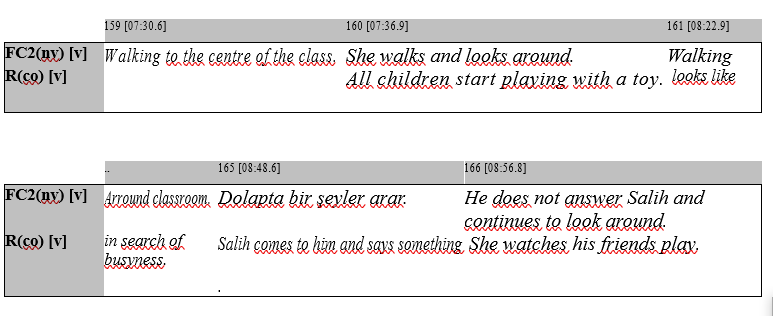


**Supplementery Excerpt 39**


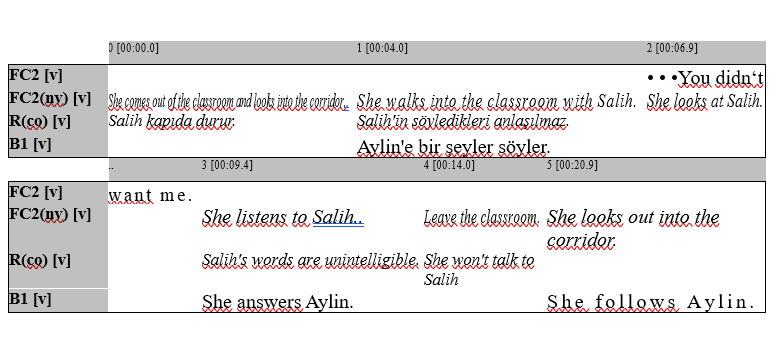


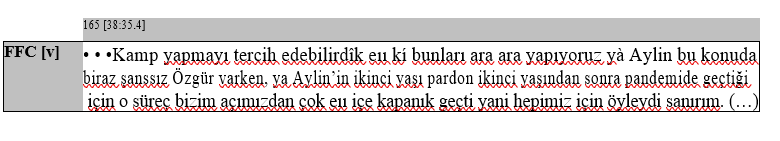
**Supplementery Excerpt 40**

*Translation: We would have preferred camping, which we do from time to time. Aylin is a bit unlucky with Özgür. Since Aylin went through the pandemic after her second year, this process was very introverted for us, I think it was like that for all of us.*

**Supplementary Excerpt 41**


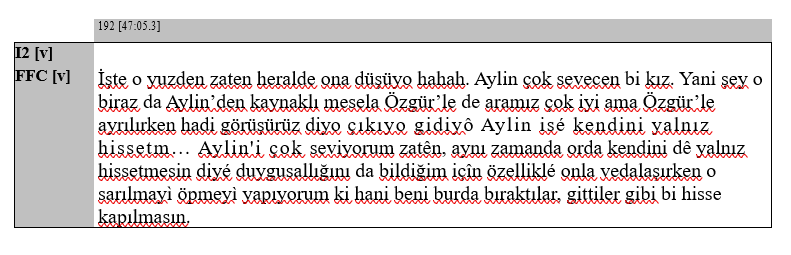


*Translation: That's why he's probably in love with her anyway, hahah. Aylin is a very affectionate girl. I mean, it's a little bit because of Aylin, for example, Özgür and I get along very well, but when he leaves with Özgür, he says "see you later" and leaves. Aylin doesn't want him to feel lonely... I love Aylin very much anyway, at the same time, because I know her emotionality, so she doesn't feel lonely there, I especially do that hug and kiss when I say goodbye to her, so she doesn't feel like they left me here, they left.*

**Supplementary Excerpt 42**


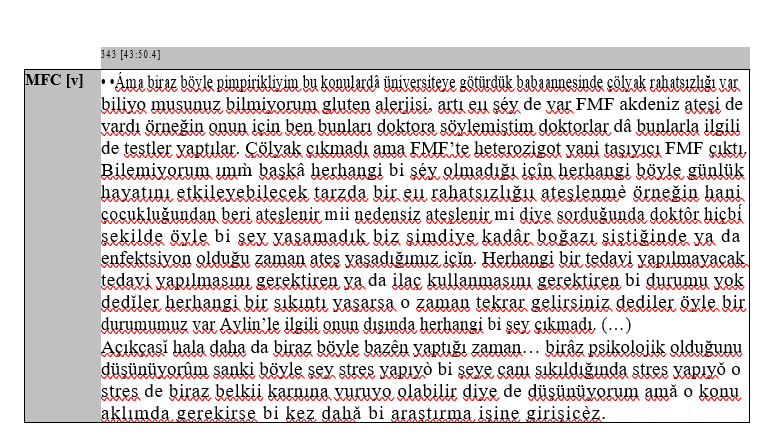


*Translation: But I'm a bit fussy, we took her to university, her grandmother has coeliac disease. I don't know if you know gluten allergy, plus FMF Mediterranean fever. There was, for example, I told the doctor about it and the doctors said and they did tests. No celiac disease was found, but I was heterozygous for FMF, so I was a carrier of FMF. I don't know, I don't know, because there is nothing else. An illness that can affect your life, such as a fever, you know. When the doctor asked if he had had a fever since childhood and if he had had a fever for no reason, the doctor said that he had never had a fever. If his throat is swollen, we've never seen that before. If there is an infection for which we have a fever. There's no treatment. There is no condition that needs treatment or medication. You said that if the teacher had any problems you would come back. We have a situation with Aylin, but there's nothing else. (...) To be honest, it is still a bit psychological... when he does that sometimes... I think it's like he's stressed about something like that. He stresses when he's bored with something. I also think it's stress that hits your stomach a little bit, maybe it's amǎ that subject. I think we'll do some more research if necessary.*
